# Investigation of possible solubility and dissolution advantages of cocrystals, I: Aqueous solubility and dissolution rates of ketoconazole and its cocrystals as functions of pH

**DOI:** 10.5599/admet.661

**Published:** 2019-04-05

**Authors:** Jaydip M. Vasoya, Ankita V. Shah, Abu T.M. Serajuddin

**Affiliations:** 1 Department of Pharmaceutical Sciences, College of Pharmacy and Health Sciences, St. John’s University, 8000 Utopia Parkway, Queens, NY 11439, USA (Jaydip M. Vasoya: jaydip.vasoya15@stjohns.edu); 2 Current address: Freund-Vector Corporation, 675 44^th^ street, Marion, IA 52302, USA (Ankita V Shah: Ankita.Shah@freund-vector.com)

**Keywords:** Ketoconazole, cocrystal, solubility, pH effect, intrinsic dissolution rate, solid phase, phase conversion

## Abstract

Since there are conflicting reports in the literature on solubility and dissolution advantages of cocrystals over free forms, we systematically studied solubility and intrinsic dissolution rates of a weakly basic drug, ketoconazole, and its cocrystals with fumaric acid and succinic acid as functions of pH to determine what advantages cocrystals provide. pH-solubility profiles were determined in two different ways: one by lowering pH of ketoconazole aqueous suspensions using HCl, fumaric acid and succinic acid, and the other by adjusting pH of cocrystal suspensions using respective coformer acids or NaOH. Similar pH-solubility profiles were obtained whether free base or cocrystals were used as starting materials to determine solubility. With the addition of fumaric and succinic acids to aqueous suspensions of free base to lower pH, the maximum solubility (pH_max_) was reached at pH ~3.5-4.0, below which the solubility decreased and cocrystals formed. The solubility, however, continued increasing when HCl was added to ketoconazole suspension as no cocrystal or salt was formed. During determination of cocrystal solubility, a conversion to free base was observed when pH was raised above pH_max_. Thus, pH-solubility profiles of cocrystals resembled solubility profiles commonly encountered with salts. Above pH_max_, both free base and cocrystal had similar solubility under identical pH conditions; the solubility of cocrystal was higher only if the pH differed. In contrast, intrinsic dissolution rates of cocrystals at pH>pH_max_ under identical bulk pH were much higher than that of free ketoconazole since cocrystals had lower microenvironmental pH at the dissolving surface, where the solubility was high. Thus, cocrystals of basic drugs can potentially provide higher dissolution rates under intestinal pH conditions.

## Introduction

Crystalline forms of acidic and basic drugs or their salts have traditionally been used as active pharmaceutical ingredients (API) [1]. During the past two decades, cocrystal, which is another class of crystalline drug substance, has gained much interest in the pharmaceutical field for potential development as API [2-5]. The US Food and Drug Administration (FDA) defined cocrystals as “crystalline materials composed of two or more different molecules, typically active pharmaceutical ingredient (API) and co-crystal formers (‘coformers’), in the same crystal lattice” [6]. To exclude solvate, hydrate, salt, etc., from such a definition, an international panel of experts defined cocrystals as “solids that are crystalline single-phase materials composed of two or more different molecular and ionic compounds generally in a stoichiometric ratio, which are neither solvates nor simple salts” [7]. The cocrystals differ from salts as no acid-base proton transfers are involved in cocrystal formation. They are usually formed by hydrogen bonding or other electrostatic interactions like π-π interaction, halogen bonding, etc., between two species, and, thus, cocrystals may form even between neutral molecules [8]. It is believed that cocrystals dissociate into individual molecular species when they dissolve in aqueous media.

According to Kale et al. [9], the development of cocrystals into viable drug products could be challenging because of such factors as ‘lower than expected supersaturation solubility, safety of coformer, difficulties in the manufacturing of high-dose drugs, polymorphism, atypical behavior of cocrystal in the formulation, difficulty in IVIVC, and so forth. Despite such challenges, there is active research on the development of cocrystals as potential drug products, and to the best of our knowledge, three of them have already received regulatory approvals. The first is ipragliflozin L-proline, a 1:1 cocrystal of the selective SGLT2 inhibitor ipragliflozin with L-proline, which received approval in Japan in 2014 for the treatment of Type 2 diabetes (Suglat® Tablet; Astellas) [10,11]; to the best of our knowledge, it is not yet available in the USA or European countries. The second is sacubitril/valsartan, which is a 1:1 cocrystal between sacubitril sodium, a neprilysin (NEP) inhibitor, and valsartan sodium, an angiotensin receptor 1 (AT1) blocker (Entresto® Tablet; Novartis); it was approved by the FDA and the European Medicines Agency (EMA) in 2015 for the treatment of long-term heart failure in adult population. The third is etrugliflozin L-PGA, which is a 1:1 cocrystal between etrugliflozin, another SGLT2 inhibitor, and L-pyroglutamic acid; it received FDA and EMA approvals in, respectively, 2017 and 2018 for the treatment of Type II diabetes (Steglatro® Tablets; Merck/Pfizer). There is not much information in the published literature on physicochemical properties of these cocrystals and what advantages they provide over those of their parent molecules.

There is the potential that a cocrystal may have higher solubility than that of the parent chemical entity. However, the solubility improvement was possibly not the primary reasons why the three marketed cocrystals were developed. In case of ipragliflozin L-proline (Suglat®), the chemical structure of ipragliflozin indicates that, by itself, it could be water-soluble since it is a glycoside with a -F and several –OH groups [10], and, therefore, the cocrystal was possibly developed for certain other advantages than the solubility improvement. In case of sacubitril/valsartan (Entresto®), both components were active moieties, and it was not a typical cocrystal between drug and therapeutically inactive coformer. Also, since both components of the cocrystal were water-soluble sodium salts, the primary advantage of cocrystal formation in this case appears to be the improvement of physicochemical properties of materials by crystallizing two otherwise amorphous or less crystalline API together as one highly crystalline drug substance [12]. Etrugliflozin is a Biopharmaceutics Classification I (BCS I) drug having good solubility and good permeability throughout the gastrointestinal pH range [13] and, therefore, its conversion to etrugliflozin L-PGA cocrystal (Steglatro®) could also be for reasons other than the solubility enhancement.

Although, as mentioned above, there may be different reasons behind the development of cocrystals, much of the recent interest in this area appears to be the potential advantage of using cocrystals to improve solubility and dissolution rate of poorly water-soluble drugs [14,15]. However, there are mixed reports in the literature on the actual increase in aqueous solubility that may be attained by cocrystal formation. Kozak et al. [16] reported that the solubility of a neutral compound ethenzamide in water increased from around 0.80-0.85 μg/mL to around 1.20-1.30 μg/mL by cocrystallization with glutaric acid, malonic acid and maleic acid. Although this could be 1.4-1.6 time increase in solubility, considering the extremely low aqueous solubility of ethenzamide and the cocrystals, the increase may not be of biopharmaceutical significance. Indeed, the cocrystallization did not increase dissolution rates of ethenzamide, and, rather, cocrystals exhibited lower dissolution rates in water than the parent compound. Although possibly not suitable for neutral compounds, it could be possible that the cocrystal formation may be especially advantageous to basic and acidic compounds in cases where they do not normally form salts. Stanton and Bak [17] determined comparative solubility of ten cocrystals of an experimental basic drug, AMG 517 (p*K*_a_ 0.68), with ten acids (p*K*_a_ 3.07 to 4.20) in simulated fasted intestinal fluid (FaSIF; 5 mM sodium taurocholate and 1.5 mM lecithin in pH 6.8 phosphate buffer) and observed that, as compared to the free base solubility of 5 μg/mL, the solubility of the cocrystals ranged from 1 to 21 μg/mL. Thus, the solubility of the compound either decreased or only marginally increased. In another study [18], the solubility of a basic drug, lamotrigine, in water was 0.154 mg/mL, while the solubility of its cocrystals with glutaric acid, sorbic acid, propionic acid and acetic acid increased to 1.10, 1.12, 2.17 and 2.55 mg/mL, respectively, and, thus, there were 7 to 17 times increase in aqueous solubility. However, at the same time, pH of the aqueous slurries during the determination of solubility decreased from 6.8 for the free base to, respectively, 5.2, 5.1, 5.0 and 4.9 for the four cocrystals. Since lamotrigine has a p*K*_a_ value of 5.7, the increase in its aqueous solubility by the formation of cocrystals may be attributed partly or fully to the pH change and the resultant protonation of the compound rather than cocrystal formation. Similarly, Gao et al. [19] observed a four-fold increase in solubility of adefovir dipivoxil, a basic drug, in water from 0.79 mmol/L to 3.33 mmol/L due to cocrystal formation with saccharin that may also be attributed to the decrease in pH of saturated solutions from 6.7 to 3.1. Cheney et al. [20] found that when there was no difference in pH of saturated solutions, there was also no significant difference among solubilities of free base and cocrystals forms of drug. In this case, solubilities of lamotrigine-methyl paraben, lamotrigine-nicotinamide and lamotrigine-nicotinamide (monohydrate) cocrystals at 1:1 ratios and that of the lamotrigine free base in water, all having pH of ~5.5, were, respectively, 0.21, 0.30, 0.23 and 0.28 mg/mL, indicating no significant difference in solubility due to cocrystal formation. In another study, Ràfols et al. [21] compared dissolution rates of a zwitterionic compound, ciprofloxacin, and its cocrystal with resorcinol under different pH conditions and observed that the parent compound dissolved faster at pH 2, while the cocrystal had somewhat higher dissolution rates in the intermediate pH conditions of 4, 5 and 5.5. However, at the intestinal pH condition of 6.5, both ciprofloxacin and the cocrystal had much lower and essentially similar solubility and dissolution rates irrespective of whether the studies were conducted in buffer or fasted state simulated intestinal fluid (FaSSIF), indicating that there were no solubility and dissolution advantages of cocrystal formation under intestinal pH conditions.

The above review of published studies does not show any major difference in equilibrium solubility between cocrystal and neutral chemical entity or between cocrystal and free base in aqueous media, and where a difference was observed, it could possibly be explained by the change in pH of solutions. There are, however, several reports where differences between kinetic solubility of cocrystals and free forms of drug were observed when dissolution rates of powders were studied. It was observed that there were initial increases in drug concentrations, which decreased with time as the free base forms precipitated out [17,22,23]. This is analogous to the supersaturation of amorphous drugs prior to their conversion to crystalline forms [24]; when the solubility reaches equilibrium, any difference between solubility of cocrystal and free drug form diminishes or disappears.

In contrast to the above-mentioned experimental findings, several investigators have demonstrated through a combination of experimental studies and theoretical calculations that cocrystals can indeed have major solubility advantages over their respective free forms, sometimes by several orders in magnitude [22,23,25,26]. The higher solubility of cocrystals as the function of pH in these studies were obtained by applying a new 3-step process: first, drug and coformer concentrations in equilibria with cocrystals were measured at pH values of interest; second, cocrystal solubility and solubility product (*K*_sp_) were determined from the measured equilibrium concentrations; and, third, the pH dependence of cocrystal solubility is theoretically calculated from *K*_sp_ of cocrystal, p*K*_a_ values of drug and coformer, and corresponding solubility equations [22]. Based on such theoretical calculations, Chen et al. [22] observed much higher solubility for 1:1 cocrystals of ketoconazole, a basic drug (p*K*_a_ 2.94 and 6.51) [27], with fumaric acid, succinic acid and adipic acid in the pH range of 4 to 7 as compared to that of the free base in the same pH range. Martin et al. [28] also observed 75 to 100 times higher solubility of ketoconazole cocrystals with fumaric acid, succinic acid and adipic acid in water than that of ketoconazole free base. However, in the latter case, the pH values of the slurries of cocrystals in water decreased from the initial 5.8 to the range of 3.4 to 4.1, where the solubility of the drug could be much higher and thus be responsible for the observed difference between cocrystal and drug solubility.

The inconsistencies between above-mentioned experimental and theoretical findings indicate that much additional work is necessary to elucidate what advantages cocrystals may have over their free forms with respect to solubility and dissolution rate. Therefore, the present investigation has been undertaken to systematically determine solubility and dissolution rates of a basic drug, ketoconazole, and two of its cocrystals with fumaric acid and succinic acid as functions of pH. Although not many studies on pH *versus* solubility of cocrystals have been reported in the literature, there are numerous reports on the effect of pH on solubility of acids, bases and their salt forms available [29-32]. There are also numerous reports on higher dissolution rates of salts as compared to their respective free base or acid forms due to change in pH and increased drug solubility in diffusion layers of solids during dissolution testing [29,32,33]. It was, therefore, of interest to determine whether cocrystals could influence drug solubility and dissolution rate analogous to salts and, if any differences in performance of salts and cocrystals existed, what were those differences. Based on these considerations, primary objectives of the present study with ketoconazole and its cocrystals were the following: (a) determine equilibrium solubility of free base and cocrystals as functions of pH to ascertain what differences exist between the solubility of two forms, (b) determine solid phases in equilibria with solutions to study whether any conversion from cocrystal to free base or *vice versa* occur during solubility vs. pH determination, (c) determine possible similarity and dissimilarity between pH vs. solubility of cocrystal with pH-solubility profiles of salts reported in the literature and, in particular, whether any pH_max_ (pH of maximum solubility) exists during the interconversion of cocrystal and free base like that with salts, and (d) determine comparative intrinsic dissolution rates of cocrystals and the free base at different pH conditions to ascertain what role cocrystal formation plays in dissolution and whether there are any impacts of microenvironmental pH at the dissolving solid surface on the dissolution rate.

## Experimental

### Materials

Ketoconazole was purchased from Alfa Aesar, a Thermo Fisher Scientific company (Ward Hill, MA, USA) and used as received. Succinic acid and fumaric acid were purchased, respectively, from VWR Life Sciences (Radnor, PA, USA) and Fluka Analytical (Fisher Scientific, Pittsburg, USA). All other solvents and reagents used in this investigation were of analytical grade or better. Distilled water was used, as necessary, for all experiments.

### Preparation of ketoconazole cocrystals

Cocrystals were prepared by modifying solvent evaporation methods reported earlier [28]. In a typical experiment for the preparation of ketoconazole-succinic acid cocrystal, 2 millimoles of ketoconazole (1063 mg) and 2.2 millimoles of succinic acid (260 mg) were dissolved in 4 mL of 3:1 v/v chloroform-methanol solvent mixture by stirring on a hot plate magnetic stirrer at 60-65 °C. The solution was then kept under vacuum for solvent evaporation and thereby cocrystal precipitation. After complete evaporation of solvent, the dried material was washed with 5 mL of methanol to remove any unconverted free drug and coformer. The washed cocrystals were dried in a vacuum oven at 50 °C to remove any residual methanol. Dried cocrystals were analyzed using differential scanning calorimetry (DSC), powder X-ray diffraction (PXRD) and Raman spectroscopy. The purity of cocrystals or the presence of any unconverted ketoconazole or succinic acid with cocrystals were assessed by DSC, PXRD and Raman spectroscopy patterns. A similar method was also used for the preparation of ketoconazole-fumaric acid cocrystal where 2 millimoles of ketoconazole (1063 mg) and 2.2 millimoles of fumaric acid (255.4 mg) were dissolved in 5 mL of 3:2 v/v chloroform-methanol mixture. Multiple batches of the two cocrystals were prepared for use in the course of the present investigation and the quality of materials was found to be consistently similar.

### Differential scanning calorimetry (DSC)

The DSC scans were recorded using the Q200 differential scanning calorimeter (TA instruments, Wilmington, DE, USA). Accurately weighed samples (4-6 mg each) were taken into Tzero aluminum pans and sealed with hermetic lids with pin holes. Samples were heated at the rate of 10 °C/min from 5 °C to 350 °C under nitrogen flush at the rate of 50 mL/min. Data acquisition was performed using TA analysis software.

### Powder X-ray diffraction (PXRD)

Powder X-ray diffraction analysis was conducted using Shimadzu XRD-6000 diffractometer (Shimadzu, Kyoto, Japan), equipped with Ni filter and monochromatic Cu-Ka radiation source. The diffractometer was operated with a copper anode tube at the generator voltage and the current of 40 kV and 30 mA, respectively. X-ray patterns of all solid samples were recorded between 5° to 50° two-theta angles at the rate of 2° per minute. All PXRD patterns were analyzed for characteristic peaks of ketoconazole and its cocrystals.

### Raman spectroscopy

Solid samples as well as the excess solids present in suspensions during the determination of aqueous solubility were analyzed by Raman spectroscopy. Raman spectra were generated with the RXN1 system (Kaiser Optics, Ann Arbor, MI, USA) using fiber optic probe emitting 785 nm laser with the total power of 400 mw. Data acquisition was performed using IC Raman 4.1 software from Mettler Toledo (Columbus, OH, USA). For *in situ* analysis of solids suspended in aqueous media, aliquots of suspensions were transferred into small aluminum cups and then exposed to laser beam through non-contact Raman PhAT Probe (Kaiser Optics) in dark. After acquisition of the spectrum, the sample was poured back into the vial and the shaking was continued.

### HPLC analysis

Drug concentration was analyzed by reverse phase high pressure liquid chromatography (HPLC) (HP 1100 series, Agilent Technologies, Wilmington, DE). The mobile phase consisted of 3:1 ratio of acetonitrile: acetate buffer, where, for the preparation of acetate buffer, 0.04 M sodium acetate trihydrate solution was prepared in distilled water, pH was adjusted to 4.5 using glacial acetic acid, and to 1000 mL of this solution, 2 mL of triethyl amine was added. For HPLC analysis, C18 column (Agilent ZORBAX Eclipse XDB columns, Agilent Technologies, Santa Clara, CA) was used with 0.75 mL/min flow rate, 230 nm wavelength, and 25°C temperature.

### Determination of pH vs solubility profiles

In a ‘white paper’ published by an international panel of experts, Avdeef et al. [34] reported that a systematic determination of solubility by taking into consideration such factors as equilibration time, stirring rate, equilibrium pH, temperature, compositions of aqueous media (buffers, ionic strength, etc.), characteristics of solid phase, and so forth, is critically important for accurate measurement of solubility of drug substances and especially when the drug substances are ionizable. The composition of solid phases in equilibria with aqueous media may also change depending on the buffering agents used [35]. For these reasons, we have undertaken systematic determination of aqueous solubility of ketoconazole, an ionizable drug, under different experimental conditions, as described below, by taking into considerations the recommendations made in these publications.

#### Ketoconazole solubility by pH adjustment with HCl

The pH-solubility profile of ketoconazole was generated by adjusting pH of its suspension in water using different concentrations of HCl solutions (0.001 to 1 M), as necessary, to obtain the desired pH. In a typical experiment, 5 mL of distilled water was taken in a 20-mL glass vial with a small amount of ketoconazole (5-10 mg), and the vial was shaken in a water bath shaker (Innova 3100, New Brunswick Scientific Co., Edison, NJ) at 25 °C. Multiple vials were prepared using different concentration of HCl solutions (0.001M to 1M) so that different pH values could be attained, and, when needed, more amount of free base was added to maintain excess solid in the vials. Ketoconazole solubility was measured at enough number of pH points by repeating above mentioned procedure as exact replicates of pH point is experimentally difficult. The pH of the suspension was measured at 4 and 24 h and concentrations of ketoconazole at these time points were determined by filtering 0.5 to 1 mL of aliquots through poly(vinylidene fluoride) (PVDF) filter with 0.45 micron pore size and analyzing filtrate by HPLC. Additional HCl solution and excess ketoconazole were added to the suspension in vial and shaken for another pH point, and the process was repeated until enough points on the pH scale were obtained. More than one suspension in different vials were used to generate a complete profile.

#### Cocrystal solubility by adjusting pH with coformer acids and NaOH

Cocrystal solubility at different pH values was determined using solutions of coformer acid (succinic acid or fumaric acid) to lower pH of aqueous suspensions and by adding NaOH solutions to adjust higher pH. Excess amount of cocrystal was dispersed in 5 mL of distilled water in a 20 mL glass vial, and when the cocrystal suspension reached a specific pH after equilibration in a water bath at 25 °C by shaking, coformer solutions (4 mg/mL fumaric acid or 50 mg/mL succinic acid) were added to respective cocrystal suspensions to lower pH, and the vials were shaken for additional 96 h at 25 °C in water bath. After equilibration, the concentration of ketoconazole at the attained pH was determined by withdrawing 0.5-1 mL of aliquot for filtration through 0.45 micron filter, followed by HPLC analysis. For higher pH, different volumes of 0.1 M NaOH solution was added to cocrystal suspensions and shaken for 96 h, and the drug concentration was determined according to the method described above. The solid phase in equilibrium with a suspension was analyzed *in situ* intermittently by Raman spectroscopy and by DSC at the end of the experiment for any conversion of cocrystal to free base.

#### Cocrystal solubility in buffered/non-buffered media

Cocrystal solubilities were determined at pH 1 solution (0.1 M HCl), pH 2 HCl buffer), pH 5 acetate buffer and pH 8 phosphate buffer. Buffers were prepared according to the United States Pharmacopeia (USP) procedures. For the determination of solubility, 5 mL of each buffer was equilibrated with excess cocrystal in a 20 mL glass vial and shaken for 96 h in a water bath shaker at 25 °C, 0.5-1 mL of samples were withdrawn at 24 and 96 h, and pH of the solution and concentration of ketoconazole were measured. Aliquots were filtered by 0.45-micron PVDF filter and analyzed by HPLC. The solid phase was analyzed by Raman spectroscopy.

#### Ketoconazole solubility by adjusting pH with fumaric acid and succinic acid

pH-solubility profiles of ketoconazole were also generated using the two coformer acids, e.g., succinic acid and fumaric acid. A series of solutions with different concentrations of acids were prepared and 3 mL quantities of these solutions were distributed in separate glass vials. For the determination of ketoconazole solubility *versus* pH using succinic acid, the concentration of acid ranged from 100 μg/mL to 50 mg/mL, while, in case of fumaric acid, the concentration of acid ranged 50 μg/mL to 2 mg/mL; the difference in acid concentration was due to difference in aqueous solubility of acids, fumaric acid being much less soluble. To each of these solutions, excess amounts of ketoconazole (~10-15 mg more than the expected solubility at a specific pH) was added and kept for shaking in a water bath shaker at 25 °C for up to 96 h. Aliquots of suspensions (0.5-0.7 mL each) were, however, withdrawn from each vial after 4, 24 and 96 h of shaking for analysis of drug concentration by HPLC according to the method described earlier. For the analysis of solid phase, contents of the vials were transferred to 3-mL Eppendorf tubes and centrifuged using Beckman GS 6R centrifuge at 2000 RPM for 5 min. After centrifugation, the supernatants were removed and solid contents were dried in a vacuum oven at 50 °C, which were then analyzed by DSC and Raman spectroscopy.

#### Solubility of 1:1 physical mixtures of ketoconazole with different coformers

Aqueous solubility was also determined for 1:1 molar physical mixtures of ketoconazole with each of the coformers (succinic acid and fumaric acid). In this study, different quantities of the mixtures (12 mg to 350 mg) were added to different 20 mL glass vials, and then 5 mL of water was added to each vial. Thus, the volume of water in each vial remained the same and only the amount of material added differed. The vials were shaken in the water bath shaker at 25 °C and 250 RPM to achieve equilibrium solubility. pH of the suspensions differed because there were different amounts of acids added in the same volume of water, and all the acids dissolved in water. Samples were withdrawn from each vial at 4, 24 and 96 h to measure drug concentration, as described earlier. The solid phase of the vials was analyzed using Raman spectroscopy to determine the identity of equilibrium species. From some of the vials, solid contents were separated by centrifugation, dried in a vacuum oven, and then analyzed by DSC.

### Intrinsic dissolution rate

Intrinsic dissolution profiles of ketoconazole free base and its cocrystals were determined using an intrinsic dissolution apparatus (Agilent Technologies, Santa Clara, CA) with 0.5 cm^2^ exposed surface area at 25 °C. For each determination, 100 mg of powder was compressed into pellet by Carver press at 1 ton pressure for 10 seconds. The apparatus with the exposed surface of the material was immersed into 250 mL of dissolution medium and rotated at 200 RPM. pH of dissolution media during the intrinsic dissolution testing were maintained by two different methods: in one case, unbuffered media were used where either 0.1 M HCl or 0.1 M NaOH were added to keep pH constant at 3, 4 or 5 (±0.15), and in the second case, pH 3, 4 and 5 phthalate buffers prepared according to the USP were used as dissolution media to keep pH constant. Two mL of aliquot was withdrawn at each of 5, 15, 30, 60, 90 and 120 min time points, filtered by 0.45-micron PVDF filter, and analyzed by HPLC for drug concentration.

## Results and Discussion

### Ketoconazole cocrystals

**Figure 1.**
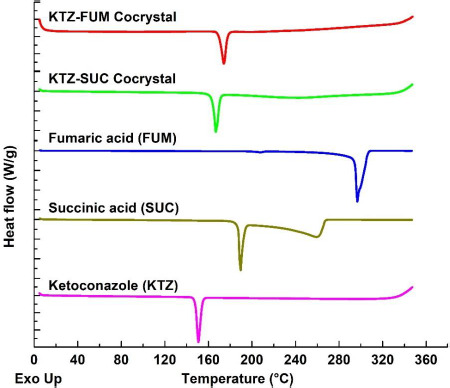
Comparative DSC scans of ketoconazole, succinic acid, fumaric acid, and ketoconazole cocrystals with fumaric acid and succinic acid.

**Figure 2.**
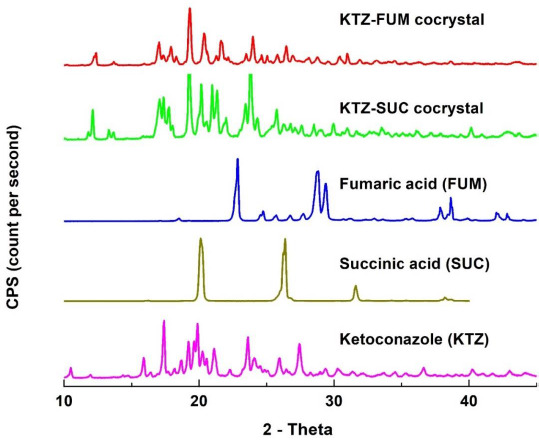
Comparative powder X-ray patterns of ketoconazole, succinic acid, fumaric acid, and ketoconazole cocrystals with fumaric acid and succinic acid.

**Figure 3.**
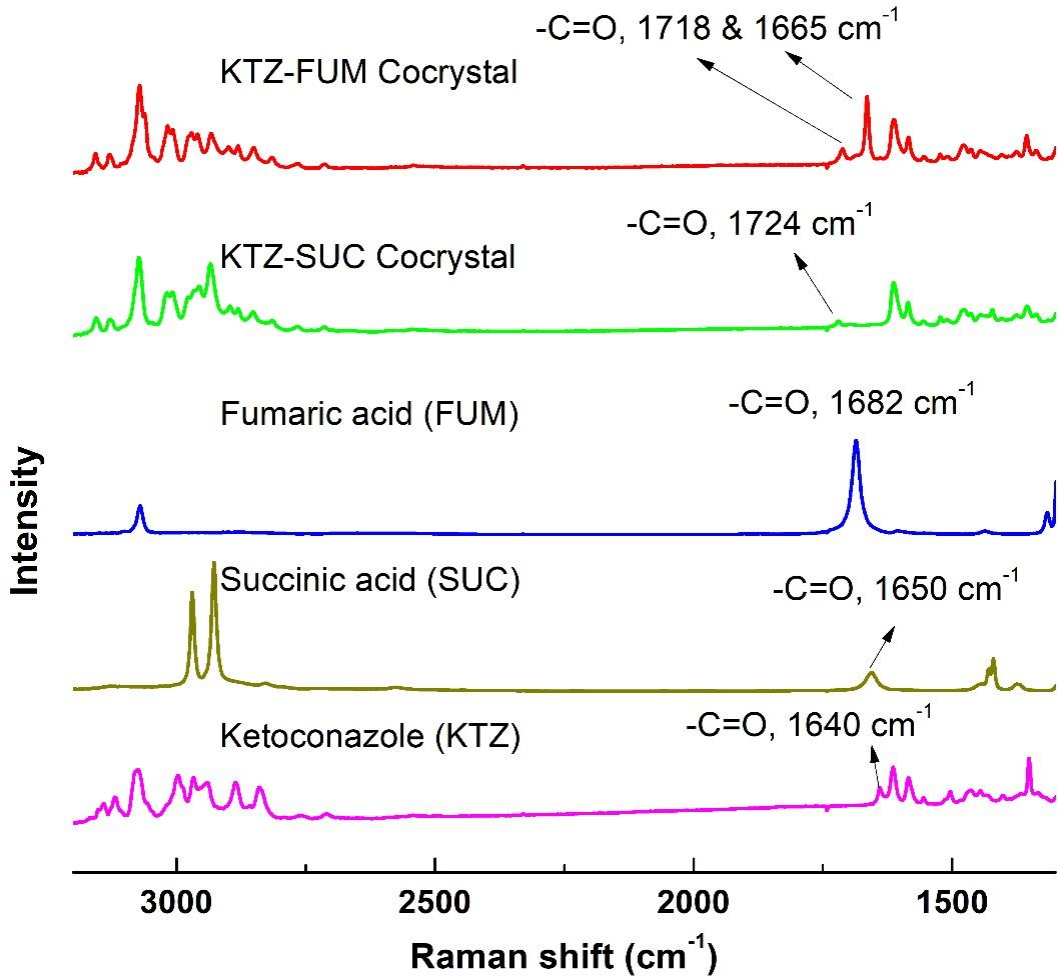
Comparative Raman spectral scans of ketoconazole, succinic acid, fumaric acid, and ketoconazole cocrystals with fumaric acid and succinic acid.

Ketoconazole-fumaric acid and ketoconazole-succinic acid cocrystals prepared by the solvent evaporation method in the present investigation were analyzed by differential scanning calorimetry (DSC) and powder X-ray diffraction (PXRD) to assess their crystallinity and purity. DSC scans of two cocrystals as well as those of parent compounds (ketoconazole, fumaric acid and succinic acid) are shown in Fig. 1, and the corresponding PXRD patterns are given in Fig. 2. Ketoconazole showed a melting endotherm at 150 °C, while ketoconazole-fumaric acid and ketoconazole-succinic acid cocrystals showed melting endotherms at 170 °C and 167 °C, respectively; these values are in agreement with those reported elsewhere in the literature [28]. The relatively sharp endotherms indicated that the cocrystals were highly crystalline, and the absence of any separate peaks for ketoconazole alone or free acids in DSC scans confirmed the absence of any impurity due to the presence of unconverted ketoconazole or coformers in cocrystals. The PXRD patterns in Fig. 2 showed characteristic peaks for ketoconazole cocrystals and their parent materials, indicating that the cocrystals are highly crystalline. Raman spectra in Fig. 3 also demonstrated that all the materials have distinct spectral patterns that are different from each other.

Ketoconazole is a weakly basic drug having two p*K*_a_ values of 2.94 and 6.51 [27]. It formed cocrystals with weak organic acids such as succinic acid and fumaric acid. Succinic acid has p*K*_a_ values of 3.93 and 5.30 for its first and second ionization, respectively, while fumaric acid has p*K*_a_ values of 2.74 and 4.17 for its first and second ionization, respectively. Childs et al. [36] postulated that a p*K*_a_ difference of greater than 3 between a basic drug and its conjugate acid (Δp*K*_a_ >3) favors salt formation rather than cocrystals, while the cocrystal formation is favored when Δp*K*_a_ is <3. The Δp*K*_a_ for ketoconazole and succinic acid is <3 and, therefore, the formation of cocrystal was expected. Although the p*K*_a_ difference between ketoconazole and fumaric acid is >3, a cocrystal, and no salt, was formed. The cocrystal formation could be due to hydrogen bonding between acetyl group of ketoconazole and one of the acid groups in succinic acid or fumaric acid. The second carboxyl groups in the acids could also form hydrogen bonds with imidazole N-atom of ketoconazole [28]. As reported by Martin et al. [28] for ketoconazole-fumaric acid pair, hydrogen bonding is favored instead of proton transfer due to spatial crystal environment in ketoconazole-fumaric acid cocrystals. They also observed that the structure of ketoconazole-succinic acid cocrystal completely resembles that of ketoconazole-fumaric acid cocrystal.

Raman spectra shown in Fig. 3 also indicate cocrystal formation. Ketoconazole has a characteristic peak at 1640 cm^-1^ for -C=O stretch while Raman spectra of succinic acid shows a peak at 1650 cm^-1^ for -C=0 (carbonyl group) of acid [37,38]. In the ketoconazole-succinic acid cocrystal spectrum, the -C=O peak shifts to 1724 cm^-1^ because of hydrogen bond formation between one of the carboxylic acid groups of succinic acid and the -C=O group of ketoconazole. This change in Raman spectrum of ketoconazole-succinic acid cocrystal shows the formation of hydrogen bond. In this cocrystal, one more hydrogen bond formation occurs between the second carboxyl group of succinic acid and nitrogen of imidazole group of ketoconazole. Due to the participation of nitrogen of imidazole (ketoconazole) in hydrogen bond formation, a shift in N=C—H stretch from 3113 cm^-1^ to 3131 cm^-1^ could be observed in the cocrystal as compared to the ketoconazole spectrum. In the ketoconazole-succinic acid cocrystal, the four-member ring formation involving two ketoconazole and two succinic acid moieties was observed [28], which is supported by two hydrogen bonds linking all four molecules in a circuit network.

Similarly, Raman spectrum of fumaric acid shows peak at 1682 cm^-1^ for the carboxylic acid group that shifts towards a higher wave number of 1718 cm^-1^ due to hydrogen bonding with -C=O group of ketoconazole in the cocrystal. The ketoconazole -C=O peak shifts from 1640 cm^-1^ to 1665 cm^-1^ due to hydrogen bond formation with the cocrystal. Also, a hydrogen bond is formed between the second carboxylic acid group of fumaric acid and nitrogen of imidazole group in ketoconazole. Thus, ketoconazole-fumaric acid cocrystal forms a similar 4-member ring structure as that of the ketoconazole-succinic acid cocrystal.

### pH versus solubility of ketoconazole base

**Figure 4.**
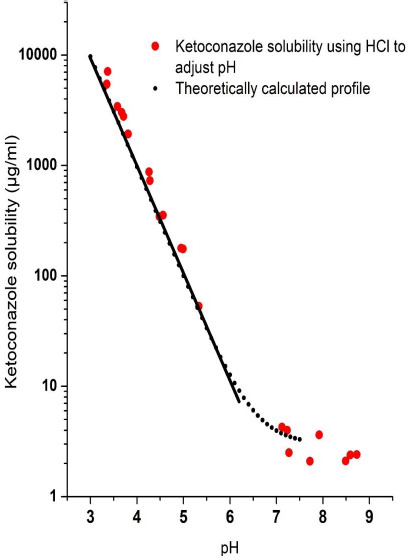
Ketoconazole pH-solubility profile generated by using hydrochloric acid to adjust pH.

For a comparison of solubility of the basic drug, ketoconazole, with that of cocrystals, the pH-solubility profile of ketoconazole was determined using HCl to adjust pH. The results are tabulated in Table 1, and they are also plotted in Fig. 4 for visual observation of the nature of change in solubility as a function of pH. The intrinsic solubility of the ketoconazole free base, i.e., the solubility of the nonprotonated species, at pH higher than 8 was found to be around 2 μg/mL, and the solubility increased exponentially as the pH was decreased gradually in the range of approximately 5.5 and 3, as indicated by the linearity of the semi-logarithmic plot in Fig. 4. The highest solubility of 148.3 mg/mL (not shown in figure) for ketoconazole at pH 3.1 was measured in the present study, when the solution turned orange. Any further reduction in pH to increase solubility by still keeping the solution in equilibrium with a solid phase was not possible; the added ketoconazole dissolved if the pH was decreased by adding concentrated HCl and the pH went back to around 3 when more solid ketoconazole was added to the solution. Although there is a report that the preparation of ketoconazole dihydrochloride salt having very high solubility and the melting point of 229 °C is feasible [39], neither the monohydrochloride salt nor the dihydrochloride salt precipitated out during the pH-solubility study in the present investigation. Attempts to crystallize ketoconazole hydrochloride salts from anhydrous methanolic solutions in separate studies were also negative as they yielded amorphous materials.

As mentioned earlier, ketoconazole has two p*K*_a_ values of 2.94 and 6.51, and the increase in solubility of the compound plotted in Fig. 4 is obviously due to the effect of the p*K*_a_ of 6.51, since any significant effect of the p*K*_a_ of 2.94 may not be observed in the pH range studied. Therefore, the solubility data are plotted in the figure according to the Equation (1), where *S*_T_ is the total solubility at a particular pH and [B]_s_ is the intrinsic solubility of the free base [31], which in case of ketoconazole was taken as 2 μg/mL.


(1)

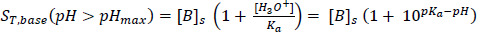



The linearity of the semi-logarithmic plot of solubility as a function of pH in the pH range of ~6.0 to ~3.5 showed that the increase in solubility follows the classic Henderson-Hasselbalch equation. There was no deviation from the linearity of the graph until the pH decreased below 3.5 since there was no effect of the second p*K*_a_ value on the solubility of ketoconazole at higher pH. The nonlinear increase in solubility around pH 3 in the semi-logarithmic plot could be due to second protonation of the molecule because of the effect of the second p*K*_a_ value.

### pH versus solubility of ketoconazole cocrystals

**Figure 5.**
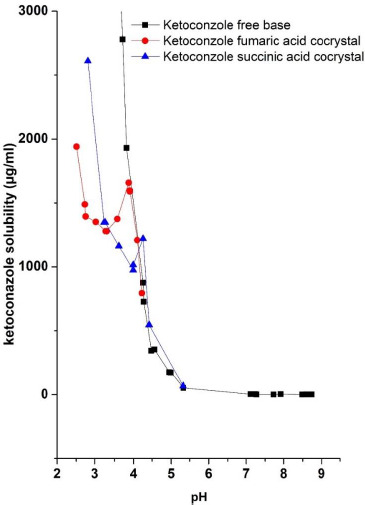
pH versus solubility profiles of ketoconazole-fumaric acid and ketoconazole-succinic acid cocrystals determined by using respective acidic coformers at pH lower than the pH of saturated solutions of cocrystals in water and by using NaOH to obtain higher pH. The solubility of the ketoconazole free base determined by adjusting pH with HCl is also plotted for reference.

The solubility of ketoconazole cocrystal as a function of pH was determined by adjusting the pH of a saturated solution of cocrystal to a lower pH by adding aqueous solutions of respective coformer and to a higher pH by adding NaOH aqueous solutions. The results are plotted in Fig. 5. When excess amounts of fumaric acid and succinic acid cocrystals were dissolved in water, pH values of the saturated solutions were, respectively, 4.1 and 4.2, and the solubilities of cocrystals were, respectively, 2.6 and 1.5 mg/mL (as ketoconazole equivalents). As examined by DSC and PXRD analyses, the solid phases in equilibria with these cocrystal solutions in water were mixtures of cocrystals and free base, the fraction of free base being relatively very small in the total solid. As shown in Fig. 5, when pH values of cocrystal solutions in water were lowered by adding respective acidic coformers, there were initial decreases in solubility, which then increased with continued addition of coformer solutions to further decrease pH. The solid phases in equilibria with these solutions were only cocrystals since no conversion to free base occurred when excess coformer was added. The mechanism of the decrease in solubility that was followed by the increase has not been elucidated. It is possible that some forms of complexes may exist between ketoconazole and coformers in solution in this pH range and the solubility profiles reflect the change in nature and solubility of such complexes.

When pH values of the saturated solutions of ketoconazole cocrystals with fumaric acid and succinic acid were increased by adding NaOH solutions, the solubility decreased for both cocrystals. The pH *versus* solubility of ketoconazole free base, which was generated by the adjustment of pH using HCl or NaOH, is also plotted in Fig. 5. A comparison of the three plots show that the solubility of the free base and the two cocrystals were identical at pH>4.1. Solid phases in equilibria with solutions during the determination of pH-solubility profiles under these pH conditions were mixtures of ketoconazole free base and cocrystals, and only at pH>5, the full conversion of the solid phase to the free base form was observed. It is apparent that when alkali is added to adjust pH, it neutralizes the acidic coformers for partial conversion of the solid phase to the free base, and when sufficient alkali is added and the pH rises above 5, all the excess cocrystals convert to the free base. It is possible that initially formed free base coated the surface of cocrystal particles and thereby only the free base was in equilibrium with the solution. These results demonstrate that the solubility of cocrystals follows the general pH-solubility profile of free base above a certain critical pH value, where the solubility decreases with the increase in pH. This is analogous to the decrease in solubility of pharmaceutical salts above the pH_max_, i.e., the pH of maximum solubility, as a function of pH [32].

### Solubility of ketoconazole cocrystals at different pH in buffered solutions

In the above section, Fig. 5 shows a decrease in solubility of ketoconazole cocrystals with the increase in pH. However, as mentioned earlier, there are reports in the literature where increased solubility of cocrystals under high pH conditions was predicted based on theoretical calculations only [20]. Since, for the development of drug products, it is necessary to experimentally ascertain solubility and dissolution rate in the gastrointestinal pH range, the solubility of ketoconazole cocrystals was also studied in buffered solutions and compared with that of the free base.

As shown in Fig. 5, there are certain limits how much lowering of pH may be achieved by adding acidic coformer solutions. For this reason, the solubility of ketoconazole cocrystals was also determined in acidic buffers to mimic gastric pH conditions. The results of these studies are tabulated in Table 2. It may be noted in this table that there were certain shifts in pH of buffers after equilibration with solids, which may be attributed to the liberation of either fumaric acid or succinic acid from cocrystals. The solubility of free base and cocrystals somewhat differed due to the pH shift. Difference in buffer compositions and ionic strength may also play roles in the observed difference in solubility in Table 2. However, overall, Table 2 shows the solubility of ketoconazole cocrystals indeed decreases at relatively higher pH conditions and the solubility of free base and cocrystals under such pH conditions are essentially similar. Such similarity in pH-solubility profiles of ketoconazole cocrystals and free base is analogous to the similarity in pH-solubility profiles of salt and free base irrespective of whether one or the other is used as starting material to determine solubility [30, 33].

### pH versus solubility profiles of ketoconazole determined by adding fumaric acid and succinic acid

It has been shown earlier in Fig. 5 that the solubility of cocrystals was influenced by pH; when pH was raised above ~pH 4.1, the cocrystals converted gradually to free base and the solubility decreased. On the other hand, when pH of the solutions was reduced by adding respective acids (coformers), the solid phase in equilibria with solutions remained unchanged and the solubility remained high. It was, therefore, of interest to determine whether a similar pH *versus* solubility relationship would exist when ketoconazole free base was used as the starting material and the pH was adjusted using fumaric acid and succinic acid. In particular, any possible conversion of ketoconazole to cocrystals by the reduction of pH during the determination of pH-solubility profile was studied.

**Figure 6.**
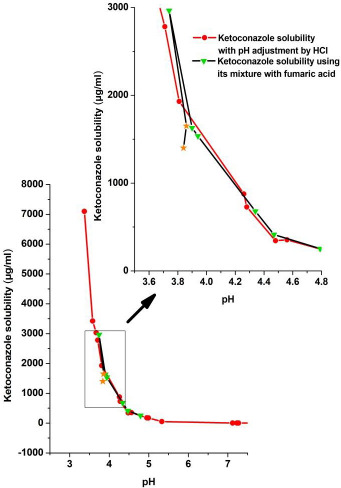
The pH-solubility profile of ketoconazole determined by using different amounts of 1:1 molar physical mixtures of ketoconazole and fumaric acid and equilibrating for 96 h. The pH-solubility profile of ketoconazole determined by pH adjustment with HCl is also superimposed for comparison. The solubility profiles from a narrow pH range (boxed region) is shown in expanded scale in the inset to indicate decrease in ketoconazole solubility after conversion to cocrystal during dissolution of the physical mixture. Asterisk symbols in the figure indicates solubility where both ketoconazole and its cocrystal with fumaric acid coexist in the solid phase.

**Figure 7.**
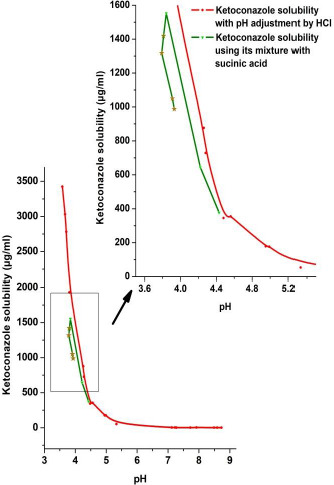
The pH-solubility profile of ketoconazole determined by using different amounts of 1:1 molar physical mixtures of ketoconazole and succinic acid and equilibrating for 96 h. The pH-solubility profile of ketoconazole determined by pH adjustment with HCl is also superimposed for comparison. The solubility profiles from a narrow pH range (boxed region) is shown in expanded scale in the inset to indicate decrease in ketoconazole solubility after conversion to cocrystal during dissolution of the physical mixture. Asterisk symbols in the figure indicates solubility where both ketoconazole and ketoconazole-succinic acid cocrystals coexist in the solid phase.

Figs. 6 and 7 give pH-solubility profiles of ketoconazole with respect to fumaric acid and succinic acid. In these studies, the solutions were equilibrated for 96 h. Both Figs. 6 and 7 were generated by dissolving different amounts of 1:1 molar mixtures of ketoconazole with, respectively, fumaric acid and succinic acid; the pH shift occurred due to the difference in amounts of acids present in added solids. Separately, solubility studies were also conducted using pre-dissolved solutions of fumaric acid and succinic acid and adding excess ketoconazole base. The results were the same whether physical mixtures were used or the free base was added to acid solutions, which indicate that acids from physical mixtures first dissolved in water and then the free base equilibrated with the solution. Therefore, we have plotted only data from physical mixtures in Figs. 6 and 7. In both of these figures, the solubility of ketoconazole increased gradually with the decrease in pH, and, after reaching peak solubilities in the pH range of 3.5 to 4.0, the solubility decreased with further addition of ketoconazole-coformer mixture or the coformer alone. Thus, it is apparent that, similar to pH_max_ (the pH of maximum solubility) generally observed in the pH-solubility profile of a free base and its salt form, the pH_max_ also exists for the cocrystal formation in aqueous media. It was confirmed by DSC analysis and *in situ* Raman analysis that, above such pH_max_, solutions in both Figs. 6 and 7 were in equilibria with the solid free base. Below pH_max_, solid phases in equilibria with solutions were mixtures of cocrystals and free base. This is because when the maximum solubility was reached at pH_max_ due the lowering of pH, the equilibrium solid phase was the free base, and, although the cocrystals crystallized out of solutions upon further lowering of pH, the undissolved free base did not readily convert to cocrystals and formed physical mixtures with cocrystals. However, upon further addition of fumaric acid or succinic acid to decrease pH, the free base in the solid phase gradually decreased and the solid converted fully to cocrystals. The pH *versus* solubility profile of ketoconazole as determined by lowering the pH of free base with HCl, as shown earlier in Fig. 4, is also partially superimposed in Figs. 6 and 7 for comparison. It is evident that until the onset of cocrystal formation (i.e, above the pH_max_), pH-solubility profiles were similar whether they were determined by adjusting pH with fumaric acid, succinic acid or HCl. The solubility is also similar to that shown in Fig. 5 by increasing pH of cocrystal solutions with NaOH. Thus, the solubility above pH_max_ was the same for free base and cocrystal and there was no special influence of cocrystal formation.

### Effect of equilibration time on ketoconazole solubility and its conversion to cocrystals

The conversion of ketoconazole to a cocrystal in the solid phase during the determination of pH-solubility profile was a slow process. As mentioned in the Experimental section, the solubility was determined by equilibrating solutions for 4, 24 and 96 h by shaking aqueous media with excess solids, and when suspensions with excess 1:1 molar mixture of ketoconazole and acids (coformers) at or below pH_max_ were equilibrated for 4h, the solid phase was the free base and no conversion to cocrystal was observed. At 24 h, the solid phase in some of the suspensions was the mixture of free base and cocrystal, and the saturation solubility was not consistently the same when the experiments were repeated, indicating that the systems did not reach equilibria. The conversion of free base to cocrystal was also influenced by how much excess solid was present in the system; the conversion to cocrystal was faster as larger amounts of excess solids were added during the determination of solubility. For these reasons, the equilibration period was increased to 96 h, when consistent results with respect to both solubility and conversion to cocrystals were obtained. It may be mentioned here that, in separate experiments, we observed that 1:1 physical mixtures of ketoconazole with coformers can be converted immediately and completely to cocrystals by co-grinding with the addition of a small amount of water. Thus, the conversion of the solid mixtures to cocrystals appears to be dependent on the proximity of ketoconazole and the coformer and how intimately they are mixed.

**Figure 8.**
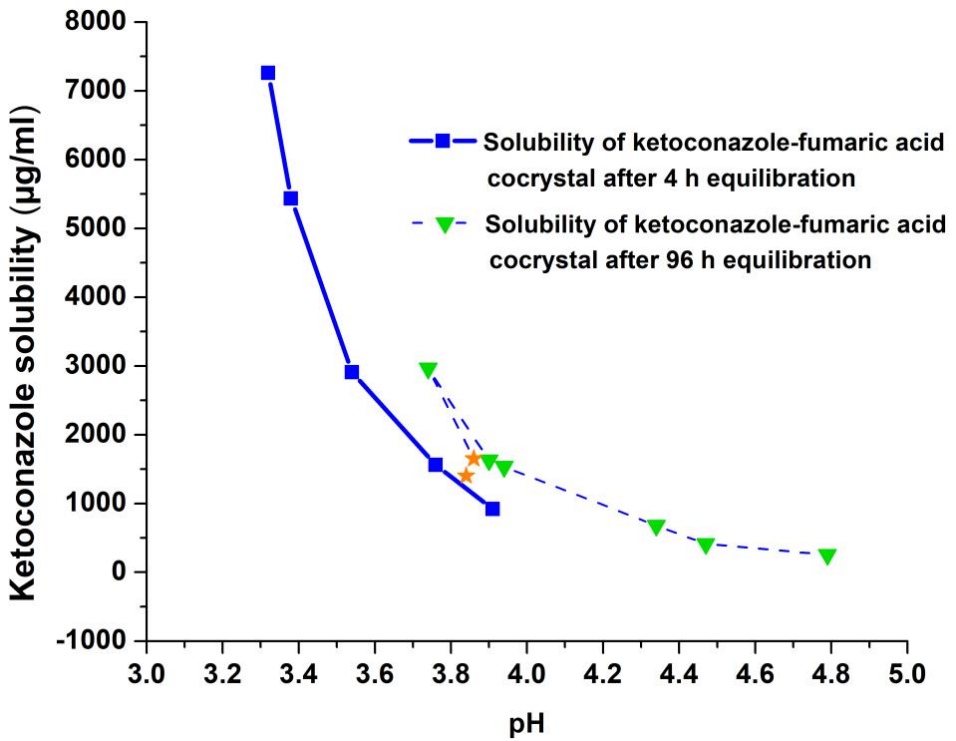
pH-solubility profiles of ketoconazole-fumaric acid physical mixtures after equilibration for 4 and 96 h. Asterisk symbols in the figure indicates solubility where both ketoconazole and ketoconazole-fumaric acid cocrystal coexist in the solid phase.

To demonstrate the effect of equilibration time on the solubility of ketoconazole, pH-solubility profiles of ketoconazole after equilibration with fumaric acid for 4 and 96 h were determined and the results are given in Fig. 8. In this figure, the solubility profile of ketoconazole after equilibration with fumaric acid at each data point for 4 h is compared with the solubility profile after equilibration for 96 h that has been shown earlier in Fig. 7. The solubility of ketoconazole increased gradually and the pH decreased when increasing amounts of 1:1 ketoconazole-fumaric acid mixture were equilibrated for 4 h at each time point. The experiment was discontinued when the solubility of ketoconazole reached about 7000 μg/mL (7 mg/mL). There was no conversion of the solid phase to cocrystals at any of the data points. In contrast, the maximum solubility of ketoconazole base observed after equilibration for 96 h was about 3000 μg/mL (3 mg/mL) at pH~3.7 and after that the solubility decreased due to the crystallization of ketoconazole-fumaric acid cocrystal. It may also be noticed in Fig. 8 that there was a small increase in pH by about 0.2 unit in the solubility profile determined by equilibration for 96 h as compared to that for 4 h. The mechanism of this effect was not investigated; however, it could be due to complexation of fumaric acid before or after nucleation into cocrystal and thus being unable to act as the free acid form.

Essentially similar effects of equilibration time were observed when succinic acid was used to adjust pH (data not shown). These results demonstrate that equilibrium solubilities of free base and their cocrystals as well as the equilibrium solid phases may differ depending on how long the systems are equilibrated. Therefore, care must be taken in interpreting any solubility differences between free bases and their cocrystal forms by taking the equilibration time into consideration.

### Intrinsic dissolution of ketoconazole free base and cocrystals at different pH

**Figure 9.**
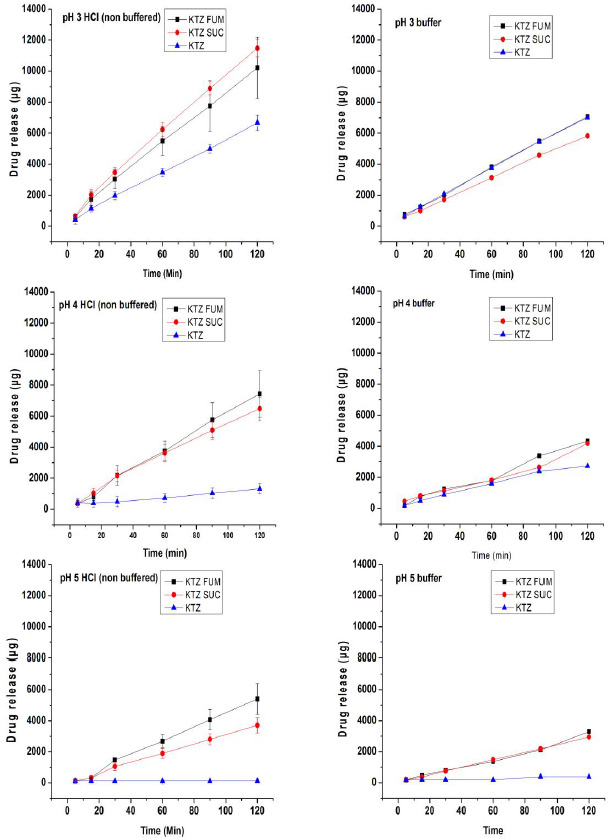
Intrinsic dissolution profiles of ketoconazole base (KTZ) and its cocrystals ketoconazole-fumaric acid (KTZ FUM) and ketoconazole-succinic acid (KTZ SUC) from a constant surface area of 0.5 cm^2^ in unbuffered media where pH was kept constant by adding HCl or NaOH, as necessary (left hand column) and in buffers (right hand column).

Fig. 9 gives the dissolution profiles of ketoconazole and its cocrystals with fumaric acid and succinic acid at pH 3, 4 and 5 from a constant surface area of 0.5 cm^2^. These pH conditions were selected because pH 3 was below the pH_max_, pH 4 was at around the pH_max_ and pH 5 was above the pH_max_.

Additionally, pH-solubility profiles in Figs. 5, 6 and 7 showed a very large decrease in solubility of ketoconazole at pH 5 as compared to those at pH 3 and 4, and it was, therefore, of interest to study whether the difference is also reflected in the dissolution rate. There are two sets of dissolution profiles in Fig. 9: the first was in unbuffered media (left hand column), where the pH of dissolution media was kept constant by adding NaOH or HCl, as necessary, and the second was in buffered media (right hand column), where no pH adjustment was necessary since the pH did not change due to the dissolution of drug.

The dissolution profiles at pH 3, 4 and 5 under pH-stat conditions in unbuffered media in Fig. 9 demonstrate that there was a great impact of cocrystal formation on the dissolution rate of ketoconazole. At all three pH conditions, dissolutions rates of cocrystals were much higher than those of the free base. Previously, comparative dissolution studies of free base and salt forms of drugs were extensively reported in the literature [30-33], where the higher dissolution rates of salts than that of their respective free base forms were attributed to the difference in microenvironmental pH conditions. Essentially, the microenvironmental pH represents the pH at the surface of solid when the diffusion layer thickness, *h*, approaches zero, i.e., *h*=0, and the solubility of drug at such a pH, and not at the bulk pH of the dissolution medium, dictates the dissolution rate of drug. The microenvironmental pH at the surface of dissolving solid is often represented as pH_h=0._ The difference in dissolution rates of ketoconazole and its cocrystals may also be explained by possible differences in their pH_h=0._

To confirm the relationship between microenvironmental pH and superior dissolution rates of cocrystals as compared to the free base, dissolution studies of cocrystals and the free base at pH 3, 4 and 5 were also conducted in buffered media, and the results are given in Fig. 9 in the right hand side along with those in the left hand side for unbuffered media. Table 3 gives their pH_h=0_ values under buffered bulk pH conditions of 3, 4 and 5 as determined by the slurry pH method described earlier in the literature [30, 40]. Essentially, in the present investigation, about 0.5 mL of buffer was added to 200 mg of powder in a small vial and mixed by vortexing, and then pH was recorded when an equilibrium was reached after 5-10 min of mixing. Although no dissolution testing was done at pH 7, pH_h=0_ values at this bulk pH condition are also given in Table 2 for a comparison with those at pH 5. It may be observed that pH_h=0_ values of ketoconazole cocrystals during dissolution in pH 3, 4 and 5 buffers were in the range of 3.13 and 4.38. The dissolution profiles in Fig. 9 are in general agreement with the ketoconazole solubility in this microenvironmental pH range, as shown in Fig. 5. For example, it may be noted in Table 3 that pH_h=0_ values of the free base and the two cocrystals in the pH 4 buffer ranged from 4.08 to 4.38, and due to the similarity in solubility in this pH range, their dissolution profiles in Fig. 9 were also similar. When the free base by itself is considered, its pH_h=0_ values at pH 3 and 4 buffers were, respectively, 4.17 and 4.38 (Table 3) and, therefore, its dissolution rates under pH 3 and 4 bulk pH conditions were also relatively high as compared to that in the pH 5 buffer (Fig. 9), where the microenvironmental pH did not change significantly and the solubility would be low.

It may be noticed in Table 3 that throughout the wide bulk pH range of 3 to 7, pH_h=0_ of the succinic acid cocrystal remained within a narrow range of 4.2 to 4.7. It is apparent that this is due to the buffering action of succinic acid present in the cocrystal, since succinic acid has two pK_a_ values of 4.2 and 5.6 and a buffer would be formed around the lower pK_a_ value. The fumaric acid cocrystals exhibited somewhat lower pH_h=0_ value since it would possibly buffer to a lower pH due to lower pK_a_ values (3.0 and 4.4). Thus, the acidity of coformers can have certain effects on the dissolution of formed cocrystals as different coformers can modulate microenvironmental pH differently.

In addition to the modulation of microenvironmental pH, the dissolution rates of cocrystals and free base may also be influenced by the supersaturation of drugs in the diffusion layer. Such supersaturation was observed earlier during the dissolution of salts [33] and free bases [30], which greatly influenced their dissolution rates. It is possible that a similar supersaturation occurred in diffusion layers during the dissolution of ketoconazole cocrystals at relatively high pH (e.g., pH 5) due to the acid-base interaction; the dissolution of free base at relatively lower pH (e.g., pH 3 and 4) would also be influenced by such an interaction. Therefore, a combination of the lowering of surface pH (pH_h=0_) and the supersaturation of drug in the diffusion layer could be responsible for high dissolution rates of cocrystals observed in pH 5 buffered and unbuffered media. In addition to pH_h=0_ values for pH 3, 4 and 5 buffers, such values for the pH 7 buffer are also given in Table 3. Since pH_h=0_ values for fumaric acid and succinic acid cocrystals at this pH were, respectively, 4.53 and 4.67, where the drug solubility is expected to be much higher than that at the pH_h=0_ of free base (pH 7.05), it is expected that dissolution rates of cocrystals at pH 7 would also be much higher than that of the free base.

Surface pH or pH_h=0_ during the dissolution of cocrystals and free base in unbuffered media could not be accurately predicted by the slurry pH method. Unlike dissolution testing, where HCl or NaOH were continuously added to keep the pH of bulk media constant, no such addition of acid or alkali was possible during the determination of slurry pH. Since the media did not have any buffer capacity, the pH in presence of excess solids in these media were essentially similar to those in water. However, the dissolution profiles in unbuffered media in Fig. 9 indicated that they were also influenced by the microenvironmental pH at the surface of dissolving solids.

Intrinsic dissolution rates of ketoconazole-fumaric acid cocrystal, ketoconazole-succinic acid cocrystal and ketoconazole free base at pH 3, 4 and 5, as calculated from the graphs in Fig. 9, are given in Table 4, which essentially confirm high dissolution rates of both cocrystals at pH 3, 4 and 5 mentioned above. In contrast, dissolution rates of the free base decreased with the increase in pH from 3 to 4, and at pH 5, the dissolution rate decreased to practically negligible level. Although individual values for dissolution rate constants at each pH may vary due to such factors as microenvironmental pH, supersaturation, etc., mentioned earlier, the results demonstrate that although there may not be significant difference in equilibrium solubility between ketoconazole free base and cocrystals at any particular pH, the cocrystal formation can greatly increase dissolution rates of basic drugs at relatively high pH conditions by modulating microenvironmental pH during dissolution testing. Consequently, for certain basic drugs like ketoconazole, dissolution rates of cocrystals could be higher than their respective free base forms. The mechanism why dissolution rates of cocrystals at pH 3, 4 and 5 in Table 4 in unbuffered media were higher than those in buffered media has not been investigated.

## Summary and conclusions

To address different issues with respect to solubility and dissolution rate of cocrystal and their free base form, we have outlined in Introduction of the paper several objectives for the present investigation. Our findings and conclusions against those objectives are summarized below:

- *Comparative solubility of free base and cocrystals as a function of pH* – pH-solubility profiles of ketoconazole cocrystals with fumaric acid and succinic showed pH_max_ (pH of maximum solubility) at pH ~ 3.5-4.0. Under pH conditions above pH_max_, the solubility profiles were identical whether fumaric acid, succinic acid, HCl or NaOH were used to adjust pH, and the profiles were also similar whether the free base or cocrystals were used as starting materials to determine solubility. At pH<pHmax, the solubility decreased in case of the addition of fumaric acid and succinic acid due to the formation of cocrystals, while the solubility continued to increase when HCl was added to lower pH as no cocrystal or salt were formed. Thus, above pH_max_ (in this case pH~4), there is no solubility advantage of cocrystal formation if the pH is maintained the same, and the solubility may differ only if pH of saturated solutions of free base and cocrystals also differ. Usually, pH values of the suspensions of cocrystals in water are lower than those of the free base in water, and, as a result, cocrystals usually exhibit higher solubility.
- *Solid phases in equilibria with saturated solutions* – During the determination of pH-solubility profiles of ketoconazole by adding fumaric acid, succinic acid or HCl, the solubility continued to increase according to the Henderson-Hasselbalch equation and the solid phase in equilibria with solutions was only the free base. After reaching a relatively high solubility at a certain pH during the addition fumaric acid or succinic acid, which may be referred to as pH_max_ or the pH of maximum solubility, the cocrystal precipitated out. Initially, the precipitate was a mixture of cocrystal and free base as the conversion of free base to cocrystal in the excess solid present was relatively slow. When the pH-solubility profile was determined by adding HCl, instead of the addition of fumaric acid or succinic acid, free ketoconazole base was always the equilibrium species as no cocrystals or salts were formed. When cocrystals were used as starting materials, instead of the free base, for the determination of pH-solubility profile, the excess solids at pH_max_ and lower were only cocrystals, and mixtures of cocrystals and free base were present when the pH was raised above pH_max_. Thus, the conversion from free base to cocrystal and *vice versa* occur, respectively, when the pH is decreased below or increased above the pH_max._
- *Similarity in pH vs solubility profiles of cocrystals and salt –* pH-solubility profiles of ketoconazole-fumaric acid and ketoconazole-succinic acid cocrystals obtained in the present investigation appeared to be similar to pH-solubility profiles commonly encountered with salts. In both cases, there are pH_max_ (the pH of maximum solubility) and the free base is the equilibrium species at pH>pH_max_. The only difference between the solubility profiles of cocrystals and salts is the nature of solid phases at pH<pH_max_; it is cocrystal in the pH-solubility profile of cocrystal, while salt is the equilibrium species in the pH-solubility profile of salt. Considering the phenomenon in another way, the two solid phases at pH<pH_max_ have certain similarities as both are complexes, the cocrystal being an acid-base nonionic complex and salt being an acid-base ionic complex. Accordingly, since both cocrystal and salt are solid complexes with definite stoichiometric ratios between basic drugs with acidic conformer or counter ion, the cocrystal may be considered as a new chemical entity similar to a salt.
- *Cocrystal vs free base intrinsic dissolution rates –* Although the solubility of cocrystals and the free base were identical at pH>pH_max_, their dissolution rates differed. Dissolution rates of cocrystals were found to be much higher than that of the free base at such pH due to the modulation of microenvironmental pH at the dissolving surface of cocrystals. For example, at pH 5, the dissolution rate of the cocrystal was higher than that of the free base, because the pH at the surface of cocrystal was lower than 5, where the solubility of ketoconazole was much higher. It was also predicted by determining microenvironmental pH that the dissolution rates of cocrystals at pH 7 would also be much higher than that of the free base. Thus, under intestinal pH conditions, the cocrystals can have better dissolution rates and, possibly, better bioavailability.

It is hoped that the results presented in this report will clarify some of the basic concepts with respect to solubility and dissolution rates of cocrystals and will provide a systematic approach to identify and select cocrystals for dosage form development. It should, however, be mentioned here that the results may be particularly applicable to basic drugs that demonstrate pH-dependent profiles in the gastrointestinal pH range. The concepts may also be applicable to cocrystals of acidic compound. However, they may not be generalized for cocrystals of neutral compounds where no significant change in solubility as a function of pH is observed. Further studies with cocrystals of relatively neutral compounds are currently underway in our laboratory.

## Figures and Tables

**Table 1:** Equilibrium solubility of ketoconazole base at different pH at 25 °C, where pH of aqueous suspensions was lowered using HCl solutions. pH and corresponding solubilities from individual vials, including repeating observations, are shown.

pH of suspension	Solubility (μg/mL)		pH of suspension	Solubility (μg/mL)		pH of suspension	Solubility (μg/mL)
**3.1**	148320		**4.28**	729		**7.23**	4
**3.35**	5457		**4.48**	345		**7.23**	4
**3.35**	5429		**4.56**	354		**7.27**	3
**3.37**	7100		**4.56**	354		**7.72**	2
**3.58**	3425		**4.95**	178		**7.92**	3
**3.67**	3031		**4.99**	175		**8.49**	2
**3.71**	2782		**4.99**	175			
**3.81**	1931		**7.12**	5			

**Table 2.** Solubility of ketoconazole (KTZ) base, ketoconazole-fumaric acid cocrystal and ketoconazole-succinic acid cocrystal in different buffer solutions along with initial pH of buffers and pH after equilibration with solids. Solubility represent average of three determinations (± standard deviation).

Initial pH and buffers used	Ketoconazole (KTZ)		KTZ-fumaric acid cocrystal		KTZ-succinic acid cocrystal	
	pH after equilibration	Solubility (mg/mL)	pH after equilibration	Solubility (mg/mL)	pH after equilibration	Solubility (mg/mL)
pH 1.1 (0.1 M HCl)	2.9*	27*	2.03± 0.01	21.54± 0.55	2.52± 0.05	23.99± 0.30
pH 2.1 HCl/KCl buffer	3.6*	3.4*	2.89 ± 0.02	3.39± 0.02	3.38 ± 0.00	3.69± 0.07
pH 5.0 acetate buffer	4.95	0.13	4.31 ± 0.02	0.60± 0.02	4.60± 0.02	0.38± 0.03
pH 7.9 phosphate buffer	7.82±0.01	0.001±0.000	7.17 ± 0.01	0.003± 0.002	7.14 ± 0.01	0.006± 0.001

*Approximate pH and solubility. As more solid materials are added, pH shifts and solubility changes.

**Table 3.** Microenvironmental pH (pH_h=0_ ) at the surface of ketoconazole (KTZ) free base, ketoconazole-fumaric acid (KTZ-FUM) cocrystal and ketoconazole-succinic acid (KTZ-SUC) cocrystal during dissolution testing in different buffers as determined by the slurry pH method. Each pH value is the average of two determinations

Buffers		pH _h=0_ (slurry pH)		
Composition	Initial pH (bulk pH)	KTZ base	KTZ-FUM cocrystal	KTZ-SUC cocrystal
pH 3 phthalate buffer	2.93	4.17	3.13	4.38
pH 4 phthalate buffer	3.96	4.38	4.08	4.19
pH 5 neutralized phthalate buffer	4.97	5.00	4.23	4.40
pH 7 phosphate buffer	6.97	7.05	4.53	4.67

**Table 4.** Intrinsic dissolution rates of ketoconazole (KTZ) free base and its cocrystals with fumaric acid (KTZ-FUM) and succinic acid (KTZ-SUC) in unbuffered and buffered media at different pH.

pH	Dissolution rate constant (μg.min^-1^.cm^-2^)					
	Unbuffered media*			Buffers		
	KTZ-FUM	KTZ-SUC	KTZ base	KTZ-FUM	KTZ-SUC	KTZ base
pH 3	164	185	106	108	91	108
pH 4	124	105	16	71	65	45
pH 5	93	62	-**	51	48	-**

*pH of bulk media kept constant by adding HCl or NaOH solutions, as necessary

**Extremely low dissolution rate (practically zero) that could not be calculated with accuracy.
